# Prefrontal hemodynamic features of older adults with preserved visuospatial working memory function

**DOI:** 10.1007/s11357-023-00862-x

**Published:** 2023-07-27

**Authors:** Tsz-lok Lee, Zihan Ding, Agnes S. Chan

**Affiliations:** 1 Department of Psychology, The Chinese University of Hong Kong, Shatin, Hong Kong; 2 Research Centre for Neuropsychological Well-Being, The Chinese University of Hong Kong, Shatin, Hong Kong

**Keywords:** Aging, Memory, Functional near-infrared spectroscopy, Cerebral hemodynamics

## Abstract

Memory decline has been observed in the aging population and is a risk factor for the later development of dementia. Understanding how memory is preserved in older adults has been an important topic. The present study examines the hemodynamic features of older adults whose memory is comparable with that of young adults. In the present study, 45 younger and 45 older adults performed the visual memory task with various difficulty levels (i.e., the items to be remembered), and their cerebral hemodynamics at each level were measured by functional near-infrared spectroscopy (fNIRS). The results showed that older adults exhibited higher activation than younger adults under more difficult but not easier levels. In addition, older adults whose performance is comparable with that of young adults (i.e., being able to remember six items) showed more right-lateralized activation. However, those unable to do so showed more left-lateralized activation. The results suggested that high-performing older adults possess successful compensatory mechanisms by recruiting cognitive resources in a specialized brain region.

## Introduction

It is well-documented that normal aging is associated with a gradual decline in cognitive function, including processing speed, selective and divided attention, episodic memory, semantic memory, visual construction skills, and executive functions [1, 2]. Working memory, a cognitive system that temporarily stores and manipulates information for performing a wide range of cognitive activities [3], is also one of the major cognitive functions that decline during the aging process [4–6]. However, it was found that some older adults can still maintain stable cognitive function [7–10]. Identifying the characteristics of these high-performing older adults may provide insight into slowing down age-related cognitive decline.

Functional neuroimaging studies have reported different activation patterns in older adults when compared to younger adults. Some studies have found lower activation in older adults, along with poorer task performance [11, 12]. Some studies have suggested that this lower activation reflects participants reaching the limits of their cognitive capacity and depleting neural resources needed to meet the challenging demand of the cognitive task [13, 14]. Conversely, greater activation was also observed in older adults [15–21]. One of the major explanations for this is attributed to compensatory processing in the aging brain. The compensation hypothesis posits that older adults spend the extra cognitive effort to compensate for their age-related deterioration in neural efficiency [22, 23]. Therefore, when task demands are subjectively low, activation is comparable between older and younger adults because the task appears effortless to both groups. However, when demands are high, greater activation is observed in older adults, indicating that they exert more cognitive effort to cope [15, 16].

Studies investigating the activation patterns of high- and low-performing older adults have consistently found differences in the extent of lateralized activation between these two groups, suggesting that they may utilize different compensation mechanisms [24–27]. However, these findings have been inconsistent, with mixed lateralized activation patterns reported across different studies. Specifically, compared to low-performing older adults, high-performing older adults were found to exhibit more bilateral activation [24], or more unilateral activation on the brain hemisphere commonly known to specialize [25, 27] (i.e., the left hemisphere for verbal tasks, the right hemisphere for visual tasks), but some studies have found the activation on the opposite side [26]. The inconsistent findings may be attributed to the varied differences in cognitive performance between the two groups across different studies. For working memory, most studies have used the 50^th^ percentile of the *n*-back task performance as an arbitrary cut-off to differentiate between the two groups [27–29]. However, this approach depends on the *n*-back task design and the performance of the recruited sample, making the differentiation between the two groups unclear. Consequently, the two groups identified in one study may not be classified in the same way in another study, which could contribute to the mixed findings reported. Therefore, an experimental paradigm with a standardized cut-off may provide a clearer sense of heterogeneity between high-performing and low-performing older adults for comparison.

The visual memory span task is a commonly employed experimental paradigm that measures visuospatial working memory [30–32]. It was modified from the Corsi Block-Tapping Test [33], a standardized neuropsychological test that has been widely used in both research and clinical settings [34–38]. In its standardized version, the examiner taps the blocks on a board in sequences, and the participant is asked to reproduce the sequence immediately. The number of blocks in the longest sequence correctly reproduced indicates the individual’s visuospatial working memory capacity. Although several versions of the visual memory span task were developed, the performance was comparable [34, 37]. In specific, while older adults with normal cognition usually obtain a score of 5 [36], those with mild cognitive impairment or dementia only obtain a score of 4 [39, 40], or even 3 [41]. By contrast, younger adults were found to obtain a score of 6 to 7 [34, 42].

Functional neuroimaging studies have suggested that the dorsolateral prefrontal cortex is one of the brain regions that play an important role in performing the visual memory span task [43, 44]. Recently, functional near-infrared spectroscopy (fNIRS) was increasingly employed in neuroimaging studies to determine task-related brain activation. fNIRS is a non-invasive optical neuroimaging technique that utilizes light in the near-infrared spectrum (650 – 950 nm) to monitor hemodynamic responses evoked by brain activities. It measures the brain tissue concentration changes of oxygenated (HbO) and deoxygenated (HbR) hemoglobin following neuronal activation [45]. It has been increasingly used in studying the hemodynamics of healthy older adults, and visuospatial working memory processing [20, 46]. Previous studies have shown that increased HbO is associated with increased cognitive demand in younger and older adults [11, 30, 47], suggesting that fNIRS is a reliable and sensitive tool in estimating cognitive effort. In addition, fNIRS studies on visual memory span tasks have found increased HbO on bilateral sides of the ventrolateral and dorsolateral prefrontal cortex [31, 32]. Besides, higher cognitive demand of the visual memory span task was associated with higher HbO in the prefrontal cortex in both younger adults [31] and older adults with mild cognitive impairment [30]. These findings suggest that the difference in the amount of cognitive effort exerted at different cognitive task loading between high- and low-performing older adults can be estimated by measuring the changes in prefrontal hemodynamics during the visual memory span task using fNIRS.

The present study aimed to understand the change in activation patterns underlying age-related working memory decline. The cognitive effort was estimated in terms of hemodynamics in bilateral prefrontal regions using fNIRS when participants were performing the visual memory span task. The present study first compared the difference in cognitive effort between younger and older adults. It was hypothesized that older adults scored lower than younger adults in the visual memory span task, together with higher HbO when the cognitive demand of the task was high, suggesting compensatory processing. The present study further explored the difference in activation patterns between high-performing and low-performing older adults. It was anticipated that two different activation patterns were observed between the two groups, representing successful and unsuccessful compensatory responses.

## Methods

### Participants

Ninety participants were recruited in this study. The younger adult group consisted of 45 undergraduate students aged from 18 to 22 years, whereas the older adult group consisted of 45 young-old to old-old adults aged from 50 to 85 years. To be eligible, participants must understand Chinese and have a normal or corrected-to-normal vision. Participants with any reported history of head injury, neurological or psychiatric disorders, or physical disabilities that affect task performance (e.g., color blindness, motor disabilities), were not eligible to participate in the present study. All participants reported no known history of cerebrovascular complications. All younger and 21 older participants reported no comorbidities that could affect vasculature. Out of the 24 remaining participants, six reported diabetes, 11 hypertension, 11 hypercholesterolemia, four hyperglycemia, two palpitations, and one coronary artery disease. In analyzing the lateralization pattern, two participants in the older adult group and three in the younger adult group were excluded because their laterality indexes exceeded at least 2 *SD* above or below the mean laterality index in each group.

All recruited older adults scored below one on the Clinical Dementia Rating Scale [48], and below a score of nine on the Functional Activities Questionnaire [49], implying no evidence of dementia. Furthermore, the memory ability of all older adults was within the normal range, defined as obtaining *Z*-scores above -1.5 based on the age- and education-adjusted normative mean on both the 10-min delayed recall score and the 30-min delayed recall score of the Hong Kong List Learning Test (HKLLT [50]), a standardized verbal memory test widely used in Hong Kong. This study was conducted according to the Helsinki Declaration of the World Medical Association Assembly and approved by the Joint Chinese University of Hong Kong-New Territories East Cluster Clinical Research Ethics Committee. All participants provided written informed consent before the study.

### Procedures

After obtaining the informed consent, all participants underwent the fNIRS session. In this session, participants were asked to perform the visual memory span task in the context of fNIRS recording. They were told to sit still and minimize head and body movements to avoid motion artifacts. Their demographic information, including age, gender, and educational level, was also collected.

### Visual memory span task

**Fig. 1:**
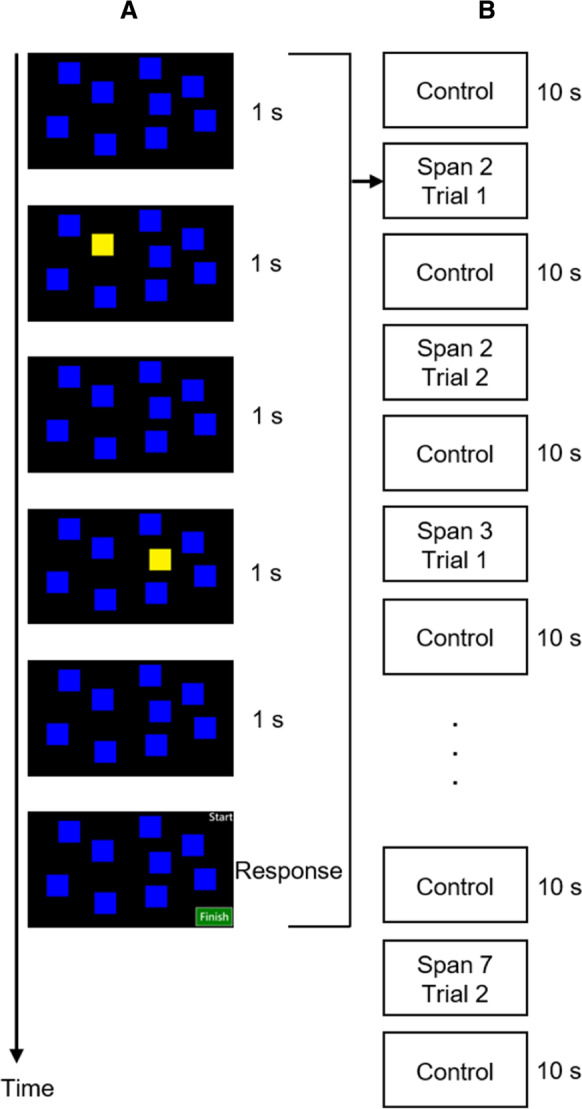
Flow diagram displaying **A** a trial with span level 2 in the visual memory span task, and **B** the flow of the whole visual memory span task

The visual memory span task paradigm was adapted from a previous fNIRS study in older adults, in which increased HbO was observed when the cognitive task demand increased [30]. In this task, each trial started with a control task period that lasted for 10 s. During this period, participants were instructed to attend to a central fixation cross that appeared on the computer screen. After the control task, nine blue square blocks appeared on the computer screen for 1 s. Next, a sequence of blocks was turned yellow, one by one each time, for 1 s. Participants were asked to memorize the sequence of the blue blocks that changed into yellow. After this encoding period, a retrieval period followed. During this period, a “start” cue appeared in the upper right corner, and a “finish” button appeared in the lower right corner. Participants were asked to reproduce the sequence by selecting square blocks in the same order as they were presented using a computer mouse, and then clicking the “Finish” button to complete (Fig. 1a). There were two trials for each span sequence, starting from a sequence of two blocks to seven blocks (Fig. 1b). There were two practice trials for participants to be familiarized themselves with the task. The stimulus presentation was performed using PsychoPy version 2022.2.4 [51]. The task score was calculated as the longest sequence length answered correctly for any one trial out of the two trials per span sequence by the participants. The completion time of each trial was also recorded. Unlike the traditional task version, the task continued even when the participants reached their longest correct span length.

### Hemodynamic measures

**Fig. 2:**
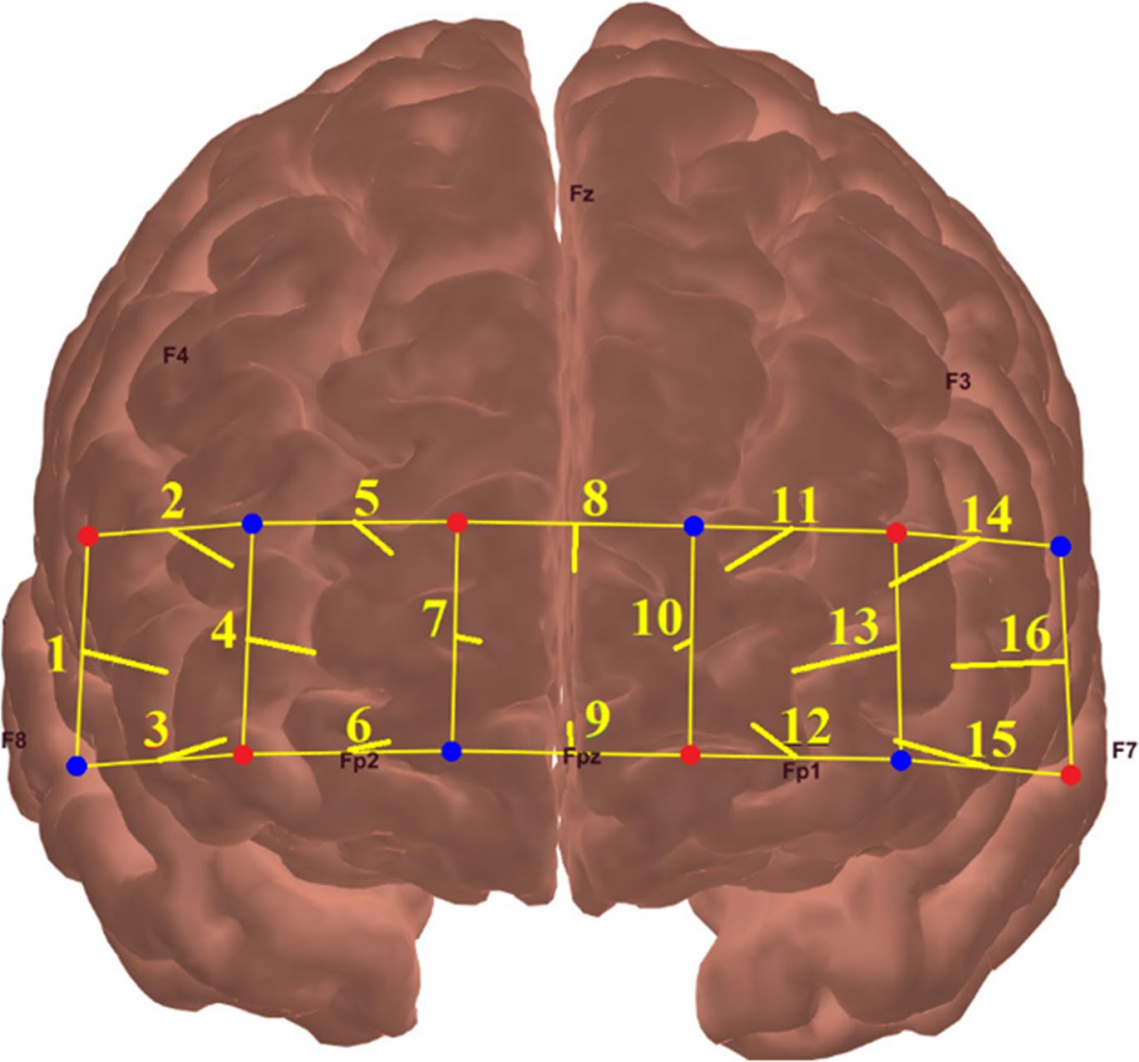
The optode, channel arrangement, and projected cortical locations for the present fNIRS system. The red and blue dots represent the source and detector probes, respectively. The yellow lines represent the 16 fNIRS measurement channels

The prefrontal hemodynamic activity during the visual memory span task was recorded using a 16-channel OEG-SpO2 system (Spectratech Inc., Tokyo, Japan). This device utilizes near-infrared light with a wavelength of 770 and 840 nm to estimate participants’ relative HbO based on the modified Beer-Lambert law [52]. The sensor consisted of six sources and six detectors arranged alternative in a 2 × 6 matrix (Fig. 2), with a source-detector separation of 3 cm. The center of the bottom probe was placed on Fpz according to the international 10/20 system. The sampling rate of this device was 12.21 Hz.

### fNIRS data preprocessing

The collected intensity data was converted into HomER3 data format for data preprocessing [53]. First, the intensity signal was converted to the optical density changes using the *hmR_Intensity2OD* function. Next, a 0.1 Hz low-pass filter was applied to remove cardiac artifacts using the *hmR_BandpassFilt* function. The filtered optical density data was then converted to HbO and HbR changes via the modified Beer-Lambert law implemented in the *hmR_OD2Conc* function, with the default value of 6 as the differential pathlength factor for both wavelengths. After that, a correlation-based signal improvement (CBSI) was applied using the *hmrR_MotionCorrectCbsi* function to improve the signal quality and reduce noise based on the negative correlation between HbO and HbR [54]. The CBSI-corrected HbO and HbR were baselines corrected with the data during the 10-s control task period before the start of the task using the *hmrR_BlockAvg* function. The baseline corrected data was averaged across all time points within each trial and then across two trials of each span. Because the HbR signal mirrored the HbO after CBSI, only the HbO data was analyzed.

Given that visual working memory is heavily reliant on the bilateral dorsolateral prefrontal cortex [55–58], the analysis focused on the fNIRS channels in this region, which were divided into the left (channel 1 – 3) and right (channel 14 – 16) prefrontal regions based on previous studies [59–62]. The HbO data in each channel within the left and right regions were averaged to improve the signal-to-noise ratio [63].

Furthermore, a laterality index was calculated to quantify the lateralization of frontal activation using the modified version of the laterality index (i.e., (A_L_ – A_R_) / (|A_L_| +|A_R_|) [64]), where A_L_ refers to the HbO changes on the left side, and A_R_ refers to the HbO changes on the right. The laterality index is a ratio measure that reflects the differences in activation levels between the two hemispheres in proportion to the overall activation level, ranging from -1 to 1. A positive value indicates left lateralization, and a negative value indicates right lateralization. The laterality index was first calculated for each homologous channel pair (e.g., channel 1 – channel 16, channel 2 – channel 14, channel 3 – channel 15). Then it averaged across pairs to represent the lateralization of frontal activation of each participant.

### Statistical analysis

First, the demographic information of the younger adult group and the older adult group was compared. The categorical variables were compared using the chi-square test. For the continuous variables, the Kolmogorov-Smirnov test was first performed to determine whether the variable violates the normality assumption. An Independent sample *t*-test was performed if normality was supported, and the Mann-Whitney *U* test was performed if normality was violated.

The behavioral performance of the visual memory span task, including the score and the reaction, was compared. Mann-Whitney *U* test was performed to examine the group difference in score. The Chi-square test was performed to determine the association between the group and the passing percentage in each span level. For the completion time, mixed ANOVA was performed to evaluate the group (younger adult group, older adult group) × span (span 2 to span 7) interaction. Spearman’s rho correlation coefficient was computed to determine the association between reaction time and span level. Post-hoc independent *t*-test was performed to compare the group difference in completion time.

For the HbO data, mixed ANOVA was performed to determine the group (younger adult group, older adult group) × region (left, right prefrontal regions) × span (span 2 to span 7) interaction. Similar to the analysis of completion time, Spearman’s rho correlation analysis was performed to evaluate the effect of span on HbO, and a post-hoc independent sample *t*-test was performed to evaluate the group difference in HbO in different span levels.

To compare the difference in activation patterns between high- and low-performing older adults, the laterality index was first analyzed with one sample *t*-test that tested against zero in each group to determine whether both groups were significantly activated during the task. An Independent *t*-test was performed to evaluate the group difference. Finally, paired *t*-test was performed in each group to determine whether the HbO was greater on one side than the other. In this analysis, only the laterality index and HbO in the span 6 levels were analyzed because this level differentiates between these two groups.

The above analysis was performed using SPSS 28.0 (IBM Corporation, Armonk, NY, USA). The significance level was set at 0.05 for all tests (two-tailed).

## Results

### Sample characteristics

The sample characteristics are presented in Table 1. The age of the older adult group (*M* = 64.1 years, range = 50.0 – 85.0 years) was significantly older than the younger adult group (*M* = 19.5 years, range = 18.1 – 21.8 years, Mann-Whitney *U* = 0, *p* < 0.001). The two groups did not differ significantly in gender (*p* = 0.052), and handedness (*p* = 0.31). It was found that the older adult group was less educated (*M* = 12.0 years, range = 1 – 19 years) than younger adults (*M* = 13.8 years, range = 12 – 16 years, Mann-Whitney *U* = 677.5, *p* = 0.006).

**Table 1:** Sample characteristics

	Younger adults (*n* = 45)			Older adults (*n* = 45)				
Variables	*M*	*SD*	Range	*M*	*SD*	Range	*U/χ2*	*p*
Age (year)	19.5	1.1	18.1—21.8	64.1	7.4	50.0—85.0	0.0	< 0.001
Gender (F/M)	32/13			23/22			3.8^a^	0.052
Education (year)	13.8	1.1	12.0—16.0	12.0	4.1	1.0—19.0	677.5	0.006
Handedness (L/R)	1/44			0/45			1.0^a^	0.31

^a^Chi-squared test was performed to examine the group difference

### Visual memory span task performance

On average, the older adult group obtained a mean score of 5.4 (*SD* = 0.9), whereas the younger adult group obtained a mean score of 6.9 (*SD* = 0.5). The task performance of the two groups was consistent with previous studies in that healthy older adults usually obtain a score of 5 on average [36], and healthy younger adults usually obtain a score of 6 to 7 on average [34, 42]. In the current sample, the younger adult group significantly outperformed the older adult group (Mann-Whitney *U* = 243.0, *p* < 0.001). Table 2 shows the percentage of participants passing the various span level. The Chi-squared test showed significant results, *χ*^*2*^(5) = 613.3, *p* < 0.001, suggesting that age was associated with different passing frequencies across spans. Specifically, it was found that most of the younger adults could remember seven spans; only four participants did not pass this level. However, 37 older adults did not pass this level. In addition, less than half of the older adults (*n* = 16) passed span level 6, and more than half (*n* = 29) failed. Further analysis was conducted to examine the HbO activation pattern associated with this difference in task performance, and the results will be shown at the end of the results section.

**Table 2:** Percentage of participants passing the span level

Span	Young Adults	Older Adults
2	100%	100%
3	100%	100%
4	100%	100%
5	100%	86.7%
6	95.6%	35.6%
7	91.1%	17.8%

**Fig. 3:**
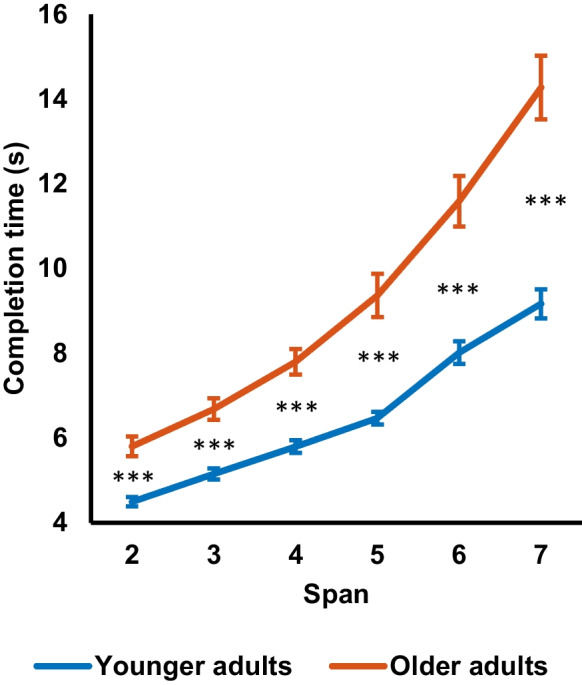
Completion time of the visual memory span task in the younger adult group (*n* = 45) and the older adult group (*n* = 45) at each span level. The error bars represent ± one standard error of the mean. The asterisks represent the significance levels of group differences in completion time. *** *p* < 0.001

The completion time at each span level was also analyzed. Mixed ANOVA was performed to evaluate the group × span interaction. The result is presented in Fig. 3. The results showed a significant main effect of span, *F*(2.6, 228.5) = 182.8, *p* < 0.001, ${\eta }_{p}^{2}$ = 0.68. It was found that the younger adult group showed significant linear (*F*(1, 44) = 393.44, *p* < 0.001, ${\eta }_{p}^{2}$ = 0.90) and quadratic trend (*F*(1, 44) = 20.60, *p* < 0.001, ${\eta }_{p}^{2}$ = 0.32) in completion time. The significant trends also appeared in the older adult group (linear contrast: *F*(1, 44) = 211.08, *p* < 0.001, ${\eta }_{p}^{2}$ = 0.83; quadratic contrast: *F*(1, 44) = 15.66, *p* < 0.001, ${\eta }_{p}^{2}$ = 0.26). Besides, there was a significant main effect of group, *F*(1, 88) = 46.0, *p* < 0.001, ${\eta }_{p}^{2}$ = 0.34, with the older adult group showing a significantly slower completion time than the younger adult group (older adults: *M* = 9.3 s, *SD* = 2.5 s; younger adults: *M* = 6.5 s, *SD* = 1.1 s; *p* < 0.001). Finally, the group × span interaction was also significant, *F*(2.6, 228.5) = 15.3, *p* < 0.001, ${\eta }_{p}^{2}$ = 0.15. The between-group comparison revealed that older adults showed significantly longer completion time at all span levels (*t* = 5.1 – 6.2, *p* < 0.001, *d* = 1.1 – 1.3). The significant interaction suggested that the group difference in completion time increased as the span level increased. Older adults had to make an extra effort to complete the task, especially when the demand was high.

### Changes in HbO of younger adults and older adults

**Fig. 4:**
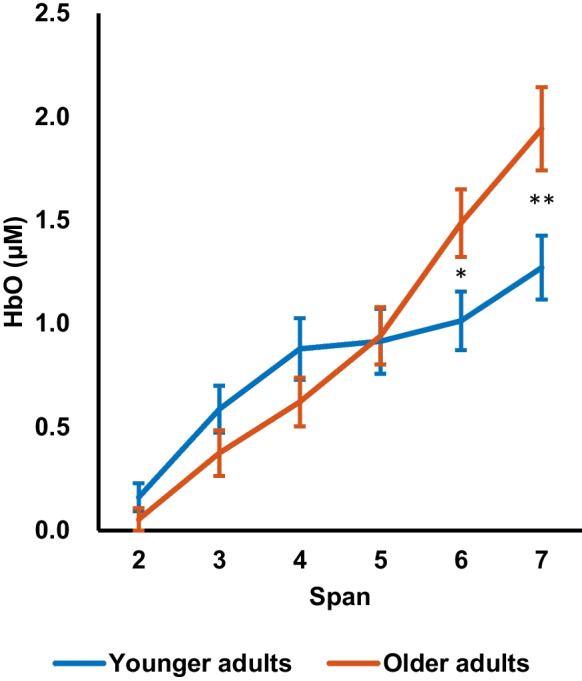
Average HbO in bilateral prefrontal regions of the visual memory task in the younger adult group (*n* = 45) and the older adult group (*n* = 45) at each span level. The error bars represent ± one standard error of the mean. The asterisks represent the significance levels of group differences in HbO. * *p* < 0.05, ** *p* < 0.01

Next, the HbO changes during the visual memory span task in each span level were compared between the two groups. Mixed ANOVA was performed to investigate the interaction between group, region, and span level in the changes in HbO. The results showed that the main effect of region, the group × region interaction, the group × span interaction, and the group × region × span interaction was not significant, *p* = 0.22 – 0.54. Therefore, the HbO was averaged across regions for further interpretation of the group × span interaction. There was a significant main effect of span, *F*(5, 84) = 38.3, *p* < 0.001, ${\eta }_{p}^{2}$ = 0.70. It was found that the older adult group showed significant linear (F(1, 44) = 114.21, p < 0.001, ${\eta }_{p}^{2}$ = 0.72) and quadratic trend (F(1, 44) = 7.59, p = 0.008, ${\eta }_{p}^{2}$ = 0.15) in HbO. These significant trends were also observed in the younger adult group (linear contrast: *F*(1, 44) = 60.08, *p* < 0.001, ${\eta }_{p}^{2}$ = 0.58; quadratic contrast: *F*(1, 44) = 6.87, *p* = 0.012, ${\eta }_{p}^{2}$ = 0.14). In addition, there was also a significant group × span interaction, *F*(5, 84) = 5.8, *p* < 0.001, ${\eta }_{p}^{2}$ = 0.26. The post-hoc comparison revealed that the older adult group exhibited a higher HbO than the younger adult group in span 6 level (old: *M* = 1.4 µM, *SD* = 1.0 µM; young: *M* = 1.0 µM, *SD* = 0.87 µM; *t*(88) = 2.4, *p* = 0.020, *d* = 0.50), and span 7 level (old: *M* = 1.9 µM, *SD* = 1.2 µM; young: *M* = 1.2 µM, *SD* = 0.94 µM; *t*(81.8) = 2.9, *p* = 0.005, *d* = 1.1, Fig. 4). The significant difference in HbO was observed in both left and right prefrontal regions. Specifically, the older adult group exhibited a higher HbO in the left region at span 6 level, *t*(88) = 2.59, *p* = 0.011, *d* = 0.55, and at span 7 level, *t*(88) = 2.78, *p* = 0.007, *d* = 0.59, and in the right region at span 7 level, *t*(88) = 2.67, *p* = 0.009, *d* = 0.56. No significant group difference in HbO changes in the other span levels (i.e., span 2 to span 5), *p* ≥ 0.15. The main effect of the group was not significant, *p* = 0.51. The results suggest that compared to the younger adult group, the older adult group exhibited a higher HbO only during trials with higher cognitive demand, indicating more effortful processing during the difficult level.

As mentioned, it was found that the education level was lower in the older adult group. However, education level was not significantly correlated with the HbO in either span 6 (*ρ* = 0.006, *p* = 0.96) or span 7 levels (*ρ* = 0.061, *p* = 0.57) in the current sample, suggesting that education was less likely to explain the significant group difference in HbO in span 6 and span 7 level.

### Activation pattern between high- and low-performing older adults

In the span 6 level, 35.6% of the older adults could remember 6 spans and 64.4% failed to do so. Using the accuracy in this level as a cut-off, the older adults were split into high-performing older adults, who have achieved similar visual memory span task performance as younger adults (i.e., answer correctly in span 6), and low-performing older adults (i.e., failed to complete span 6 correctly). There was no significant age difference between the low-performing older adults (*n* = 28) and the high-performing older adults (*n* = 15), *p* = 0.13.

**Fig. 5﻿:**
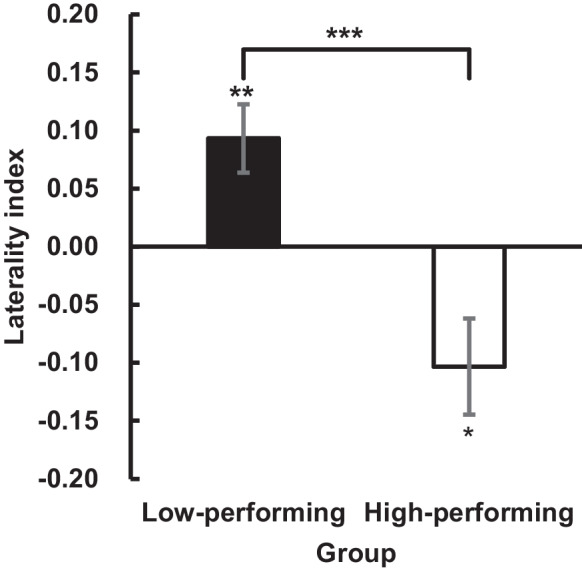
Laterality index in span 6 level in low- (*n* = 28) and high-performing older adults (*n* = 15). A positive value indicates left lateralization, whereas a negative value indicates right lateralization. The asterisks above each error bar represent the significance level of one sample *t*-test again zero. In contrast, the asterisks above the square brackets represent a significant difference between the two groups. The error bars represent ± one standard error of the mean. * *p* < 0.05, ** *p* < 0.01, *** *p* < 0.001

The difference in activation pattern was then explored between these two groups. First, the laterality index was compared (Fig. 5). One sample *t*-test showed that the low-performing older adults had a significant left lateralization, *t*(27) = 3.2, *p* = 0.004, *d* = 0.60, whereas the high-performing older adults had a significant right lateralization, *t*(14) = 2.5, *p* = 0.026, *d* = 0.47. Furthermore, the independent sample *t*-test showed that the high-performing older adults had a significantly smaller laterality index than did the incorrect group, *t*(41) = 3.9, *p* < 0.001, *d* = 1.2, suggesting that the correct group had significantly greater right lateralization than did the low-performing older adults.

**Fig. 6:**
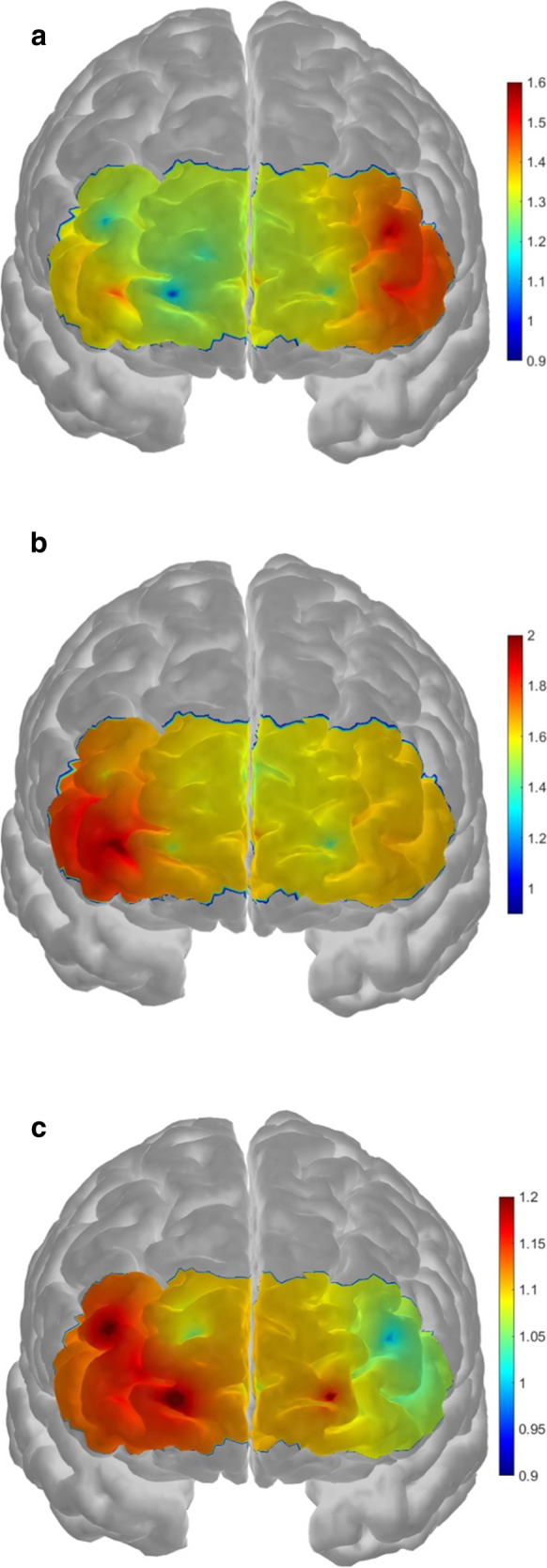
Prefrontal activation maps showing the HbO concentration changes (in µM) in span 6 level of the visual memory span task in **A** low- (*n* = 28), **B** high-performing older adults (*n* = 15), and **C** younger adults (*n* = 42). Data from individual channels were used in creating all activation maps. Red color represents greater activation, whereas blue color represents less activation. It was found that the low-performing older adults showed significantly greater activation in the left prefrontal region than the right region (*p* = 0.026), whereas the high-performing older adults showed significantly greater activation in the right region than the left region (*p* = 0.022). The younger adult group also showed significantly greater activation in the right region (*p* = 0.047)

Next, the mean HbO changes within the left and right prefrontal regions in the span 6 level were also compared. One sample *t*-test showed that both groups exhibited significantly higher HbO against zero (*p* < 0.001), suggesting that bilateral prefrontal cortices were significantly activated in both groups. Consistently with the results observed in the laterality index, the low-performing older adults exhibited greater HbO increases on the left region than the right (right: *M* = 1.3 µM, *SD* = 0.90 µM; left: *M* = 1.6 µM, *SD* = 1.1 µM; *t*(27) = 2.4, *p* = 0.026, *d* = 0.45, Fig. 6a). By contrast, the high-performing older adults exhibited greater HbO increases on the right region than the left (right: *M* = 1.8 µM, *SD* = 1.2 µM; left: *M* = 1.5 µM, *SD* = 1.0 µM; *t*(14) = 2.6, *p* = 0.022, *d* = 0.67, Fig. 6b). There was no significant between-group difference in mean HbO changes in both prefrontal regions (*p* ≥ 0.11). The results suggest that the high-performing older adults demonstrated successful compensatory processing by exhibiting significantly higher lateralized activation in the right prefrontal region.

Consistently, the high-performing older adults responded faster than the low-performing older adults at span 6 level (high-performing: *M* = 9.7 s, *SD* = 2.0 s; low-performing: *M* = 12.6 s, *SD* = 4.5 s; *t*(41.8) = 3.0, *p* = 0.004, *d* = 0.84), which provides converging evidence suggesting that the high-performing older adults were more cognitively efficient than the low-performing older adults. It is noted that both groups did not differ in terms of memory ability. Specifically, the group differences in the 10-min delayed recall (high-performing: *M* = 9.6, *SD* = 3.8; low-performing: *M* = 8.9, *SD* = 2.7; *t*(23.8) = 0.62, *p* = 0.54), and the 30-min delayed recall (high-performing: *M* = 9.1, *SD* = 3.6; low-performing: *M* = 8.7, *SD* = 2.8; *t*(43) = 0.42, *p* = 0.68) of the HKLLT were not significant. Therefore, the significant difference in lateralized activation on the right region between the high- and low-performing older adults was less likely due to group differences in memory ability.

### The activation pattern in younger adults

After understanding the activation pattern between high- and low-performing older adults, the same analysis was performed in younger adults to determine whether there was lateralization observed in the younger adult group as well. One sample *t*-test showed that the younger adult group had significant right lateralization, *t*(41) = 2.73, *p* = 0.009, *d* = 0.42. Furthermore, there was a significantly greater HbO increases on the right region than the left region in the span 6 level (right: *M* = 1.17 µM, *SD* = 0.84 µM; left: *M* = 0.98 µM, *SD* = 0.97 µM; *t*(41) = 2.05, *p* = 0.047, *d* = 0.32, Fig. 6c). Therefore, the activation pattern in the high-performing older adults was more closely resemble the younger adult group, but not the low-performing older adults.

## Discussion

The present study utilized fNIRS to investigate the age-related compensatory mechanism by measuring the prefrontal hemodynamics during the visual memory span task. It was found that older adults performed poorly as compared to younger adults. In addition, older adults showed a higher prefrontal activation than younger adults only when the cognitive loading of the trial was high (i.e., the span 6 and 7 levels, in which less than half of participants answered them correctly). The older participants demonstrated a compensatory attempt by putting much cognitive effort into coping with the high cognitive task demand, consistent with previous findings [15, 16]. More importantly, the present study explored further the difference in activation patterns associated with higher visuospatial working memory performance in older adults. It was found that high-performing older adults, who performed similarly to the younger adults, exhibited more right-lateralized activation when the cognitive demand was high. In contrast, the low-performing older adults exhibited more lateralized activation, but to the left hemisphere. Given that visuospatial working memory is commonly known as specialized in the right hemisphere [65], the significant difference in lateralized pattern suggests that high-performing older adults performed better by cognitive resources allocation to the specialized brain region. In contrast, the low-performing older adults failed to put much cognitive effort because of efficiently recruiting cognitive resources in the opposite region. Therefore, together with the shorter reaction time in high-performing older adults, commonly known as an index of cognitive efficiency, it is believed that the high-performing older adults were more cognitively efficient.

Unlike previous studies that utilized the 50^th^ percentile of the *n*-back performance as a cutoff score to differentiate the high- and low-performing older adults [27–29], the present study attempted to differentiate the two groups using the visual memory span task, which was modified from a standardized neuropsychological test used in the clinical setting. Given that younger adults usually obtained a score of 6 to 7 in the task [34, 42], the high-performing older adults were believed to preserve an intact working memory function similar to the younger adults because these older adults could correctly complete span 6. Some could even recall seven spans correctly. Therefore, the visual memory span task could impose an absolute cut-off to differentiate between the high- and low-performing older adults, making the results comparable across studies. Besides, the *n*-back task was prone to a ceiling or floor effect when the recruited sample consisted of a wide range of working memory abilities. On the contrary, the visual memory span task was found suitable for samples with different levels of working memory abilities, such as younger adults and older adults with normal cognition, mild cognitive impairment, and dementia [34, 36, 39–41]. Therefore, the visual memory span task was employed in the present study. The present findings support the use of this experimental paradigm in understanding how an individual makes his cognitive efforts in handling cognitive tasks with increasing cognitive loading.

The hemodynamic responses measured by the fNIRS rely on the neurovascular coupling mechanism, which describes the relationship between neuronal activity and blood flow [45]. A previous study has reported an age-related decline in neurovascular coupling [66]; it is unclear whether the higher HbO levels observed in older adults in the present study were due to the difference in task-related neural activity or the age-related reduction in neurovascular coupling. If the observed difference was due to the difference in neurovascular coupling, there should be a difference between older adults with preserved and declined neurovascular coupling. Assuming that older adults with comorbidities that could affect vasculature have relatively declined neurovascular coupling compared to those without [67, 68], these two groups did not significantly differ in terms of the score of the visual memory span task (*p* = 0.41), and the task-related hemodynamic response (*p* = 0.23 – 0.65). Therefore, the effect of neurovascular coupling on the task-related hemodynamic response difference between younger and older adults observed in the present study appeared small, if not negligible. Despite this, future studies may consider the age-related difference in neurovascular coupling when understanding the age effect on brain activation.

There have been conflicting results regarding the age-related changes in brain activation. While some reported an increased activation in older adults [15–18], others have reported a decreased activation [11, 12]. In addition, there has been a debate regarding the Compensation-Related Utilization of Neural Circuits Hypothesis (CRUNCH) model. This compensation model posits that older adults exhibit higher activation when cognitive task demands are low to compensate for age-related decline in cognitive processing. Furthermore, they exhibit lower activation when cognitive task demands are too high, which exceeds their cognitive capacities. Previous studies have reported findings that support this model (e.g., [69]). However, inconsistent with what the CRUNCH model predicts, the present study found that the older adults exhibited significantly higher HbO at the most difficult level (i.e., span 7), which was sufficiently challenging as only 17.8% of older participants could perform correctly. This finding is consistent with a previous fMRI study that failed to replicate the CRUNCH effect [70]. The mixed findings regarding age-related activation and different models of compensation suggest that there may be moderators that affect age-related brain activation, such as the differences in neurovascular coupling between younger and older adults, as mentioned before [66, 71].

Aging has become a serious global health issue because the population of older adults has been increasing at an accelerated rate. It was estimated that the world’s population of people aged 60 years and older will increase from 1 billion in 2020 to 1.4 billion in 2030, doubling to 2.1 billion in 2050 [72]. Therefore, developing an effective intervention that slows down the cognitive decline or maintains the cognitive function of older adults will be beneficial to relieve the social and economic burdens brought on by the aging problem. In the present study, the higher neural efficiency observed among high-performing older adults suggests the possibility of improving cognitive function by enhancing the neural efficiency of older adults. Brain stimulation techniques such as transcranial direct current stimulation [73], transcranial alternative current stimulation [74], and transcranial photobiomodulation [75] have been recently considered as potential effective interventions that alter brain functions. Future work may explore whether these methods can enhance neural efficiency and slow down age-related cognitive decline in healthy older adults.

Several limitations were noted in this study. For instance, the present study included a wide range of older participants aged between 50 and 85 years. Besides, the older adult group in the present study was assessed for possible dementia and memory impairment. Future studies may consider recruiting a more homogeneous aged group with a narrower age range and with a more comprehensive cognitive assessment differentiating participants with different levels of cognitive function (e.g., normal cognition, mild cognitive impairment, and dementia).

In conclusion, the present study demonstrated that aging is associated with deterioration in working memory and extra cognitive effort when the performing task is cognitively demanding. Furthermore, it was also found that high-performing older adults engaged in successful compensatory processing by exhibiting higher activation in the right prefrontal region, a region specialized in visuospatial working memory. Low-performing older adults, however, showed higher activation in the left prefrontal region, suggesting an unsuccessful compensatory attempt by putting much cognitive effort into an unspecialized region.

## Data Availability

The data that support the findings of this study are available from the corresponding author upon reasonable request.
